# Seed dormancy is modulated in recently evolved chlorsulfuron-resistant Turkish biotypes of wild mustard (*Sinapis arvensis*)

**DOI:** 10.3389/fchem.2015.00046

**Published:** 2015-07-24

**Authors:** Muhamet Topuz, Yildiz Nemli, Tahira Fatima, Autar K. Mattoo

**Affiliations:** ^1^Sustainable Agricultural Systems Laboratory, Agricultural Research Service, United States Department of Agriculture, Henry A. Wallace Beltsville Agricultural Research CenterBeltsville, MD, USA; ^2^Plant Protection Research Station, Department of Plant Protection, Ege UniversityIzmir, Turkey

**Keywords:** *Sinapis arvensis*, ALS inhibitor, herbicide resistance, point mutations, seed dormancy, germination, gibberellins

## Abstract

Biotypes of the broad-leaved wild mustard (*Sinapis arvensis* L.) found in wheat fields of Aegean and Marmara region of Turkey were characterized and shown to have developed resistance to sulfonylurea (chlorsulfuron), an inhibitor of acetolactate synthase (ALS). DNA sequence analysis of the *ALS* genes from two such resistant (“R”) biotypes, KNF-R1 and KNF-R2, revealed point mutations, CCT (Pro 197) to TCT (Ser 197) in KNF-R1 and CCT (Pro 197) to ACT (Thr 197) in KNF-R2; these substitutions are consistent with the presence of chlorsulfuron-insensitive ALS enzyme activity in the “R” *S. arvensis* biotypes. An additional phenotype of chlorsulfuron resistance in the Turkish *S. arvensis* “R” biotypes was revealed in the form of an altered seed dormancy behavior over 4–48 months of dry storage (after-ripening) compared to the susceptible (“S”) biotypes. Seeds of the “S” biotypes dry stored for 4 months had a higher initial germination, which sharply decreased with storage time, while the seeds of the “R” biotypes had lower germination after 4-months storage, rising sharply and peaking thereafter by 24 months' of dry storage. The “R” biotype seeds continued to maintain a higher germination percentage even after 48 months of after-ripening. The seed weight of “R” and “S” biotypes after-ripened for 4 months was similar but those after-ripened for 48 months differed, “R” seeds were significantly heavier than those of the “S” seeds. Differential seed germinability between “S” and “R” biotypes was found not a case of differential viability, temperature regimen or non-response to pro-germination hormone GA_3_. These studies are of relevance to ecological fitness of herbicide-resistant biotypes in terms of seed viability and germination.

## Introduction

Maximizing crop yields are vital for feeding the burgeoning population in the world. Major obstacles to increasing crop productivity include weed competition (as high as 100 billion dollars a year or even more), and maintaining seed vigor of the cash crop upon storage since seed germination decreases upon aging (Kocsy, 2015). For instance, wheat yield losses are proportional to increased weed density (http://www1.agric.gov.ab.ca/$department/deptdocs.nsf/all/crop1280), since the latter compete for soil nutrients, water, space and light. Weeds have therefore been a major management constraint for agriculturists, farmers, and crop biologists alike. The discovery of sulfonylureas as inhibitors of acetolactate synthase (ALS) [EC 4.1.3.18], also known as acetohydroxyacid synthase (AHAS), which catalyzes the first committed step in the biosynthesis of branched-chain amino acids (valine, leucine, and isoleucine), led to sulfonylureas-based herbicides as effective weed management tool for a number of crops including wheat (Umbarger, 1978; Tranel and Wright, 2002).

Chlorsulfuron was formulated as an herbicide and introduced into agriculture worldwide over 30 years ago to control growth of monocot and dicot weed species. Its frequent use, however, led to weed resistant biotypes, such as prickly lettuce (*Lactuca seriola* L.) (Mallory-Smith et al., 1990) and kochia (*Kochia scoparia* L. Schrad.) (Primiani et al., 1990). The resistance to chlorsulfuron was linked to point mutation(s) in the ALS gene, which prevented herbicide binding to the ALS enzyme (Mallory-Smith et al., 1990; Martinez-Ghersa et al., 2000), as well as to enhanced ability to catabolize the herbicide (Primiani et al., 1990). By 2015, 246 plant species (103 monocots and 143 dicot) are reported to have developed resistance to ALS inhibitors (Heap, 2015).

The relevance of herbicide resistance is of tremendous concern for weed management including the possibility of their altered ecological fitness in regard to their growth, competitive ability and seed production and seed germination ability (Gressel and Segel, 1978; Tranel and Wright, 2002; Vila-Aiub et al., 2005). Little information is available on the fitness of chlorsulfuron-resistant weeds, although an early report found seeds of chlorsulfuron-resistant kochia biotype to germinate faster than the susceptible biotype (Dyer et al., 1993).

Wheat is grown in Turkey in ~9.4 m ha, occupying about 45% of the total arable land (FAOSTAT Database; http://www.fao.org). The application of sulfonylurea herbicides has successfully managed weeds in Turkish agriculture (including wheat, rice, and maize cultivations) in the past for over 30 years. Among these weeds, wild mustard (S*inapis arvensis* L.) is of noted importance causing substantial yield losses in wheat even when present at low seeding rate (60 plants/m^2^) (Gillespie and Nalewaja, 1988). Another study conducted on *S*. *arvensis* at seeding rates of 54 and 108 plants m^−2^ found 12–20 and 20–56% reductions in wheat yields, respectively (http://www1.agric.gov.ab.ca/$department/deptdocs.nsf/all/crop1280).

*S. arvensis* is frequently observed in the wheat growing Aegean and Marmara region of Turkey (Boz, 2000; Topuz and Nemli, 2001). Two of its biotypes, called KNF-R1 and KNF-R2, were identified as being resistant to sulfonylurea application in the Bandırma-Balıkesir province of the latter wheat-growing region. Here, we characterize chlorsulfuron resistance in these Turkish *S. arvensis* biotypes at physiological and molecular levels, linking resistance to known mutation(s) in the *ALS* gene and a chlorsulfuron-insensitive ALS enzyme. Further, we show an additional phenotype of chlorsulfuron resistant *S. arvensis* biotypes in the form of an altered seed dormancy behavior compared to the susceptible biotypes. These studies are of relevance to ecological fitness of herbicide-resistant biotypes in terms of seed viability and germination.

## Materials and methods

### Plant material

Wild mustard (*S. arvensis*) seeds were collected from surviving weeds in wheat fields sprayed with chlorsulfuron in Bandırma-Balıkesir provinces of Marmara region (latitude 40° 19′ 6″ N and longitude 27° 58′ 40″ E), Turkey. Two resistant (R) biotypes (labeled as KNF-R1 and KNF-R2) were collected from wheat fields in Kulefli, ~4 km apart, about 8 km southwest of Bandırma airport. These fields had a history of chlorsulfuron applications for weed control for several years. Chlorsulfuron-susceptible (“S”) biotypes (labeled as MR-S biotype for herbicide resistance studies; and MR-S1 and MR-S2 for seed germination studies) were collected from ditch banks next to wheat fields from which KNF-R1 and KNF-R2 biotypes originated. Seeds of each biotype were collected from five plants, bulked, air dried at room temperature, and then stored at 4°C until use.

### Whole plant herbicide dose-response studies

Dose-response data for chlorsulfuron were generated from experiments carried out both in Turkey and the USA. The remaining experiments were performed solely in the USA. In Turkey, the experiments were carried out in open net houses covered by polyethylene at the Plant Protection Research Institute, Izmir/Bornova. The seeds were directly seeded in 20 × 20 × 20 cm pots containing turf (obtained after sieving decomposed tree leaf litter):soil:sand (2:1:1 ratio), at three plants per pot. Experiments in the USA were carried out in a temperature-controlled greenhouse at the USDA, Henry A. Wallace Beltsville Agricultural Research Center, Maryland (latitude 39° 1′ 39.35″ N and longitude 76° 55'35.40W). Seeds were sown in Metro-mix 360 (Scotts-Sierra Horticultural Products, Marysville, OH, USA) in plastic containers. At the cotyledon stage, the seedlings were transplanted into 10 × 10 × 10 cm plastic pots (1 plant per pot in quadruplicate) on the same media. Temperature regime was 21°C/16°C day/night with 16 h photoperiod supplemented with 230 μmol m^2^ s^−1^ illumination. Plants were watered daily and treated at 3–4 leaf stage with chlorsulfuron (Glean/Glean®) using a hand sprayer (Turkey) or pressurized sprayer (USA), both equipped with a flat fan nozzle (8001E), calibrated to deliver 300 L ha-1 of spray solution at 210 kPa. Chlorsulfuron was applied at rates (indicated in the text) that were 0.5–8 times the recommended application rates in Turkey (10 g a.i ha^−1^), and from 0.25 to 128 times in the USA based on a calculated minimum rate of 10 g a.i ha^−1^. The untreated controls were sprayed with water instead of the herbicide. The effects of the applied herbicides were evaluated 21 days after the treatment (DAT). The shoots were cut at soil level to collect the biomass, which was then air dried at 65°C for 72 h to determine dry weight. Plant pots were arranged in a randomized complete block design, with five replications in Turkey and four replications in USA, and experiments were repeated twice. Whole plant herbicide cross-resistance was studied with trifensulfuron-methyl (doses equivalent to 4.35, 8.7, 17.4, 34.8, and 69.6 g a.i ha^−1^; Harmony® GT, 750 g a.i kg-1, Du Pont). Chlorsulfuron resistant (KNF-R1 and KNF-R2) and susceptible (MR-S) biotypes were grown as described above and treated at the 3–4 leaf stage for cross resistance pattern for trifensulfuron-methyl. In all the experiments, untreated controls were sprayed with water. Herbicides were applied with a pressurized sprayer with a flat fan nozzle as described above.

### Acetolactate synthase (ALS) enzyme assay

Leaves were excised from the resistant KNF-R1 and KNF-R2, and susceptible MR-S *S. arvensis* biotypes, and processed for the preparation of cell-free extracts, protein precipitation with ammonium sulfate and desalting on Sephadex G-25 column as previously described (Ray, 1984). ALS [EC 4.1.3.18] activity was determined in 60% ammonium sulfate precipitated protein fraction (after desalting). Total protein content was determined by the Bradford method (Bradford, 1976). The ALS enzyme assay was carried out with slight modifications as previously described (Rashid et al., 2003). The reaction assay mixture contained, in a final volume of 500 μL, desalted (NH_4_)_2_SO_4_-precipitated protein (100 μg), 300 μL of the assay medium (83.3 mM potassium phosphate, pH 7.0, containing 167 mM sodium pyruvate,16.7 mM MgCl_2_,1.67 mM thiamine pyrophosphate and 16.6 μL FAD), and H_2_O or herbicide. The herbicide concentrations were varied from 0 to 100 nM. The reaction mixtures were incubated at 35°C for 60 min and the enzymatic reaction stopped by adding 50 μl of 6 N H_2_SO_4_. After incubation at 60°C for 15 min, 25 μl of 3N NaOH were added to each tube, and pH adjusted to 7.0. The samples were microfuged for 5 min. After adding 500 μl each of creatine (Sigma, C0780, 5.45 g l^−1^ in water) and 1-napthol (54.5 g l^−1^ in freshly prepared 2.5 N NaOH), the tubes were incubated at 60°C. The samples were microfuged for 1 min and then absorbance of the color developed was measured spectrophotometrically at 525 nm (Molecular Devices Spectra Max Plus^384^). The experiment was repeated 3 times.

### ALS gene sequencing

Genomic DNA was extracted from young leaves, excised from a total of 5 plants from each biotype, as previously described (Delaporta et al., 1983) and quantified spectrophotometrically. The *ALS* gene from MR-S, KNF-R1, and KNF-R2 biotypes was PCR amplified and the PCR products sequenced to determine the sites where mutations may have arisen. Three overlapping regions were amplified using primer sets (Tan and Medd, 2002) (Table 1) and conditions as described (Warwick et al., 2005). The PCR amplification reaction contained 100 ng genomic DNA, primers (each 0.5 μM), 200 μM dNTP, 1.5 mM MgCl_2_ and 2 μl (5 units/μl) Go Taq® Flexi DNA Polymerase (Promega cat No: M8291) in a final volume of 100 μl. PCR products were separated on 1.5% low melting agarose gels and purified using Qiaquick® Gel Purification Kit (Qiagen, 28704). Direct cycle sequencing was outsourced to MWG, North Carolina (USA). Each PCR product was sequenced with both forward and reverse primers. Nucleotide and amino acid positions were numbered based on the amino acid sequence of *ALS* from *Arabidopsis thaliana* (Sathasivan et al., 1990).

**Table 1:** **List of primers used to amplify the *ALS* gene of *S. arvensis* biotypes**.

**Primer sequence 5′ – 3′**
ALS1F TTCR^a^TCTCCCGM^a^TACGCTCCC
ALS1R CAARCTGY^a^TGCTGAATATC
ALS2F GATGTTCCTAAGGATATTC
ALS2R CTGATGYTGYCCAACACC
ALS3F GGR^a^GAAGCCATTCCTCC
ALS3R TCARTACTW^a^AGTGCK^a^ACCATC
ALS11F AGGGTTACGCTCGTTCCTC
ALS11R GAAACTTCCGGAGGCTGAG

a *I.U.P.A.C-I.U.B codes for mixed bases R (AG), M (AC), Y (CT), W (AT), K (GT)*.

### Seed preparation for germination studies

Experiments carried out in the laboratory, growth chamber and greenhouse followed completely randomized design with four replications, and were repeated twice. Seeds were stored at 4°C for the times indicated in the text. Prior to use, seeds from *S. arvensis* resistant and susceptible biotypes were surface sterilized for 1 min in 70% ethanol followed by two 5 min immersions in 0.3% sodium hypochlorite and then thoroughly rinsed with sterile water (Yajima et al., 2004). The same seed lots and germination chambers were used throughout the germination assays.

In the laboratory, seeds were placed in 12-well petri dishes with 2 seeds per well (24 seeds petri-dish^−1^) on sterile double filter paper, previously moistened with sterile water (0.6 ml well^−1^). Dormancy and viability tests were carried out in the absence and presence of gibberellic acid (GA_3_) at 0.1–10 mM. GA_3_ was first dissolved in isopropyl alcohol, and then diluted with water to achieve indicated concentrations. Controls included sterile water. The petri dishes were sealed with parafilm and stored in the dark at 22°C ± 2 °C. Germination of seeds (aged 3 and 8 years) was assessed daily, in the dark, for 14 days; germinated seeds were counted and removed. Germination was defined as any seed where the seed coat was found broken and the radicle was visible.

For experiments carried out in a temperature-controlled greenhouse (Henry A. Wallace Beltsville Agricultural Research Center, MD), seeds were sown on Metro-mix 360 (Scotts-Sierra Horticultural Products, Marysville, OH) in plastic containers (36 seeds replicate^−1^), watered daily and germination evaluated for 21 days at 12 h intervals. Temperature regime was 21°C/16°C day/night with 16 h photoperiod supplemented with 230 μmol m^2^ s^−1^ illumination. The germinated seeds were counted and removed from the containers.

The effect of different temperature regimes (5, 10, and15°C) on seed germination was carried out in a growth chamber under 16 h light/8 h dark photoperiod.

### Effect of after-ripening time on seed weight and seed germination rate

Lots of 100 seeds each were randomly taken from two resistant (“R”) and two susceptible (“S”) biotypes for measuring weight of seeds that were dry-stored (after-ripening) for 4 months and 4 years. Seeds of (“R”) and (“S”) biotypes were evaluated for germination rates in a greenhouse taking seed lots stored for 4 months, 2 years, and 4 years (after-ripening time). Seeds were sown on Metro-mix 360 (Scotts-Sierra Horticultural Products, Marysville, OH) in plastic containers (36 seeds replicate^−1^), watered daily and germination evaluated for 21 days at 12 h intervals.

### Statistical analysis

The data on percentage of seeds that germinated on each plate were statistically analyzed using SPSS 15.0 for windows. Data were logarithmically transformed and then subjected to analysis of variance to determine the significance of differences between means at the 5% level of probability using the Duncan test. For further analysis, germinating seedlings were scored and converted to mean germination percentage and the standard errors of the means calculated. Dose-response curves (for chlorsulfuron) for the whole plant bioassay were plotted and statistically analyzed by SAS (1999) using a non-linear regression (Seefeldt et al., 1995) since seeds germinated at variable rates.

## Results

### Establishing herbicide resistance in Turkish *S. arvensis* biotypes

**Figure 1:**
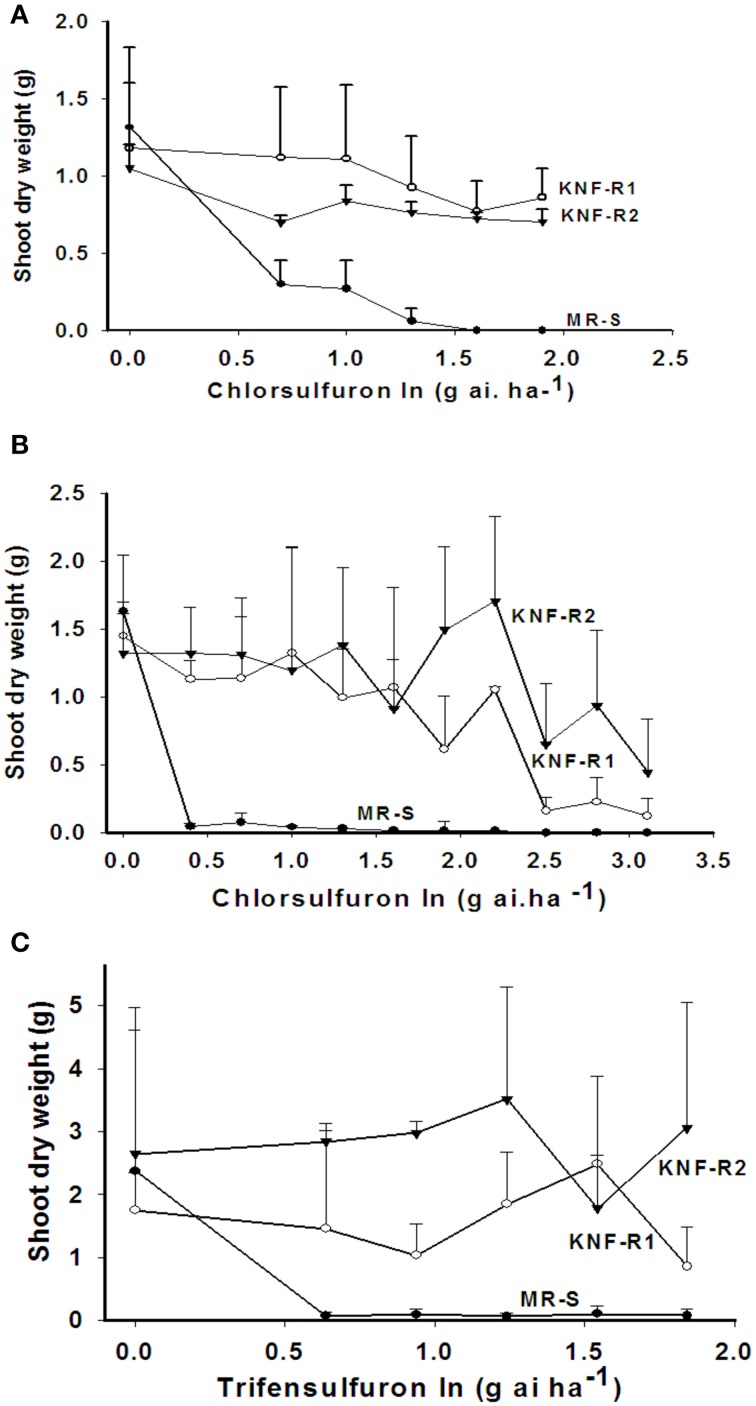
**Differential effects on the shoot dry weight (SDW) of wild mustard (*S. arvensis*) biotypes, MR-S (•), KNF-R1 (◦), and KNF-R2 (▾) in response to sprays with increasing concentrations of the ALS inhibitor, chlorsulfuron (A, B) or trifensulfuron-methyl (C) shown in log values**. **(A)** Net-house in Izmir, Turkey; chlorsulfuron rates tested (0–80 g a.i.ha^−1^). Seeds used were dry-stored for 6–9 months. **(B,C)** Greenhouse at Beltsville, MD, USA; in **(B)** chlorsulfuron rates tested (0–320 g.a.i ha^−1^). Seeds used were dry stored for 24 months. Vertical bars represent standard error [*n* = 10, for **(A,B)**; *n* = 8, for **(C)**].

The shoot dry weight (SDW), used as a measure of growth response, of the sensitive (MR-S) and resistant (KNF-R1 and KNF-R2) Turkish *S. arvensis* biotypes to application of indicated doses of chlorsulfuron was tested both in Turkey (Figure 1A) and USA (Figure 1B). In Turkish trials, the MR-S biotype was found sensitive to the lowest applied chlorsulfuron dose while both the resistant biotypes, KNF-R1 and KNF-R2, were recalcitrant to chlorsulfuron concentrations as high as 80 g a.i ha^−1^. In the USA greenhouse experiments, chlorsulfuron levels used were higher, varying a thousand-fold (0–1280 g a.i ha^−1^). The MR-S biotype was found highly sensitive to the lowest herbicide concentration of 2.5 g a.i ha^−1^ as compared to its untreated control while the SDW of KNF-R1 and KNF-R2 biotypes was not affected up to a dose of 80 g a.i ha^−1^, as also seen in the Turkish trials (compare Figure 1B with Figure 1A). However, a progressive decrease in SDW was apparent even in KNF-R1 and KNF-R2 biotypes at chlorsulfuron concentration of 160 g a.i ha^−1^ and higher (Figure 1B).

Cross resistance of the three biotypes used here was also tested using other related herbicides - trifensulfuron-methyl (thifensulfuron), metsulfuron-methyl and imazamethabenz-methyl (IMI), and an unrelated herbicide 2,4-D (Jugulam et al., 2005). The MR-S biotype was found susceptible to all of these inhibitors while the KNF-R1 and KNF-R2 biotypes were found resistant also to the trifensulfuron-methyl (Figure 1C) but not to IMI, metsulfuron-methyl, or to the plant growth regulator 2,4-D (data not shown). The dose-response curves for trifensulfuron-methyl on the SDW of the three biotypes paralleled those obtained with chlorsulfuron (compare Figures 1A,B with Figure 1C).

### Acetolactate synthase activity isolated from chlorsulfuron-resistant *S. arvensis* biotypes is insensitive to chlorsulfuron

**Figure 2:**
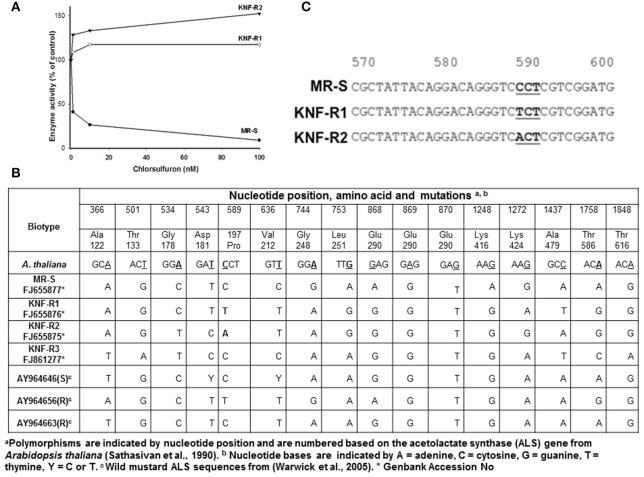
**(A)** Acetolactate synthase (ALS) activity of resistant biotypes, KNF-R1 and KNF-R2, is not inhibited by chlorsulfuron. **(B)** Nucleotide positions, amino acids and ALS gene mutations in *S. arvensis* “R” biotypes, KNF-R1 and KNF-R2, in comparison to the “S” biotype MR-S using *A. thaliana als* sequence as standard. **(C)** Representation of the sequences around the mutation in ALS gene of “R” biotypes which results in amino acid substitution. In KNF-R1, the mutation C–T represents Pro197 (**C**CT) to Ser (**T**CT) substitution while in KNF-R2 the C–A change representing Pro197 (**C**CT) to Thr (**A**CT) substitution. The ALS gene from susceptible MR-S biotype did not harbor any of these mutations.

Next, acetolactate synthase (ALS) activity was determined in the 60% ammonium sulfate precipitated protein fraction of soluble leaf extracts from KNF-R1, KNF-R2, and MR-S biotypes in the absence and presence of chlorsulfuron (Figure 2A). The ALS enzyme activity isolated from MR-S biotype was found sensitive to low concentrations of chlorsulfuron, 5 nM herbicide causing 50% inhibition of the enzyme activity. In contrast, ALS enzyme activity isolated from KNF-R1 and KNF-R2 biotypes was completely insensitive to chlorsulfuron, even at concentrations as high as 100 nM (Figure 2A). These *in vitro* data complement the *in vivo* dose response on the SDW of the three biotypes, and relate chlorsulfuron resistance of KNF-R1 and KNF-R 2 *S. arvensis* biotypes to an altered ALS protein.

### ALS gene from chlorsulfuron-resistant *S. arvensis* biotypes harbors mutations

To test our contention that the ALS protein in KNF-R1 and KNF-R2 *S. arvensis* biotypes is altered, we carried out molecular analysis by first amplifying the ALS genes of all the three biotypes using PCR, and then the PCR products were sequenced. The sequences we obtained lacked 62–64 nucleotides from the 5′ end of the gene but included all the regions of the ALS gene previously shown to have mutations that confer resistance to chlorsulfuron (Tranel and Wright, 2002). Overall, DNA sequence analysis showed a total of 16 nucleotide mutations among the *S. arvensis* biotypes, fourteen of which were determined to be silent, i.e., when translated these did not result in a change in the amino acid (Figures 2B,C). ALS gene sequences varied slightly among the three biotypes, with some polymorphism being apparent (Figure 2C). The ALS gene of Turkish *S. arvensis* biotypes showed ~1% polymorphism, similar to that detected previously (Warwick et al., 2005). Amino acid substitution of Glu290 (GAG) to Ser290 (AGT) in MR-S biotype and Gly290 (GGT) in KNF-R1 and KNF-R2 biotypes was apparent but this amino acid change in ALS gene is not known to be associated with resistance phenomenon and is highly variable (Warwick et al., 2005). Notably, the single point mutation that resulted in an amino acid substitution was found at nucleotide position 589 in the ALS gene from “R” biotypes, KNF-R1 and KNF-R2 (Figure 2C). In the KNF-R1 ALS gene, the mutation C to T represents Pro197 (**C**CT) to Ser (**T**CT) substitution while in KNF-R2 the C to A change represents Pro197 (**C**CT) to Thr (**A**CT) substitution (Figure 2C). The ALS gene from susceptible MR-S biotype did not show any of these mutations (Figure 2C).

### Chlorsulfuron-resistant Turkish *S. arvensis* biotypes are altered in seed dormancy behavior

Seed dormancy, germination, and emergence are important agronomic characteristics of weeds, which play a key role in agricultural productivity, determining competition and success of a staple crop and its weeds in an agroecosystem (Koger et al., 2004; Weitbrecht et al., 2011). Occasional reference to a possible link between herbicide resistance and seed dormancy has appeared in the literature but data are less dense. Therefore, we tested whether newly evolved chlorsulfuron resistance of *S. arvensis*, associated with altered ALS gene/enzyme, has any impact on the seed germination ability as compared to the sensitive biotypes.

**Figure 3:**
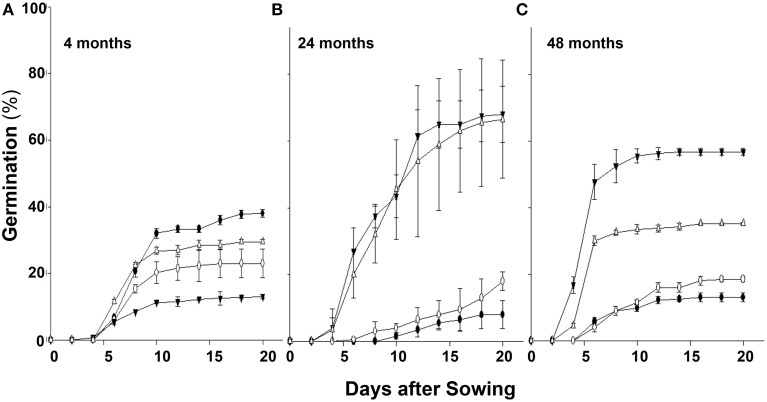
**Cumulative seed germination (%) in relation to progressive emergence of *S. arvensis* chlorsulfuron-resistant “R” [KNF-R1 (∇), KNF-R2 (▾)], and susceptible “S” [MR-S1 (•) and MR-S2 (◦)] biotype seeds**. Seeds used were dry stored for: **(A)**, 4 months; **(B)**, 2 years (24 months); **(C)**, 4 years (48 months). Vertical bars represent standard error (*n* = 6).

Over a few years during which these studies were conducted, we consistently observed variability in the germination rate of seeds of chlorsulfuron susceptible (S) vs. resistant (R) *S. arvensis* biotypes. Therefore, we carried out controlled, long-term experiments (in the USA) and compared seed germination rates of *S. arvensis* (“R”) and (“S”) biotypes after a period of 4 months, and after 2 and 4 years of dry storage. The seeds of “S” biotypes, MR-S1 and MR-S2, dry stored for 4-months had, respectively, a germination rate of 38.0 and 23.0% (Figure 3A), which decreased to 8 and 18% at 2 years (Figure 3B), and after 48-months' dry storage it was 13 and18.5% (Figure 3C). In contrast, the resistant biotypes, KNF-R1 and KNF-R2, showed an opposite germination trend to that of “S” biotypes. The germination rate of 4-months dry stored seeds of R1 and R2 biotypes was, respectively, 12.5 and 30.5% (Figure 3A), which increased to 68.0 and 56.7% at 2 years of storage (Figure 3B), and by 4 years of dry storage period it was 66.5 and 32.2% (Figure 3C). It is noted here that the seeds of “R” biotypes dry stored for 4 years germinated 24 h sooner than the “S” biotypes.

**Figure 4:**
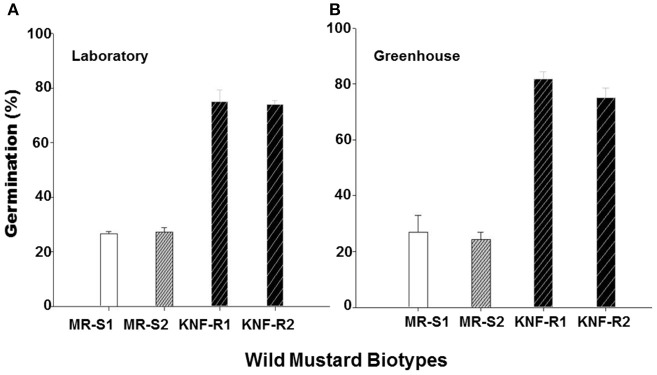
**Cumulative seed germination (%) of *S. arvensis* chlorsulfuron-resistant “R,” KNF-R1 and KNF-R2, vs. susceptible “S” biotypes, MR-S1 and MR-S2, under controlled laboratory (A) and greenhouse conditions (B)**. In both tests, seeds used had been dry stored for 2–3 years. Vertical bars represent standard error [*n* = 12 for **(A)**; *n* = 6 for **(B)**].

To further confirm the results from experiments carried out in the greenhouse, we compared germination of seeds from the “S” and “R” biotypes, dry stored for 2-3 years, at room temperature in the laboratory and at 22°C in the greenhouse. In the laboratory experiments, significantly higher percentage (74–75%) of seeds from “R” biotypes, KNF-R2 and KNF-R1, germinated compared to low % germination (26.9–27.1%) of “S” biotype counterparts, MR-S2 and MR-S1 (Figure 4A). A similar germination pattern was observed under controlled greenhouse conditions where germination of “R” biotypes was 81.9 and 75.0% in KNF-R1 and KNF-R2, respectively, compared to respective 26.8 and 24.3% in MR-S1 and MR-S2 (Figure 4B).

### Different seed germination potential of “S” and “R” *S. arvensis* biotypes is not altered by suboptimal temperature regime

**Figure 5:**
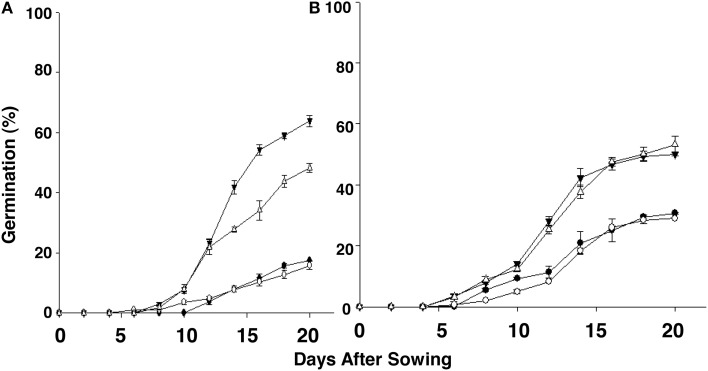
**Emerging progression of chlorsulfuron-resistant “R,” KNF-R1 (∇) and KNF-R2 (▾), and susceptible “S,” MR-S1 (•) and MR-S2 (◦), *S. arvensis* biotypes sowed in a growth chamber held constant at 10°C (A) and 15°C (B)**. Cumulative seed germination (%) is presented as supplemental material (Supplemental Figure 1). Seed lots used had been dry-stored for 30–36 months. Vertical bars represent standard error (*n* = 6).

We next investigated if low temperature regime—5, 10, and 15°C—altered the germination potential of the “S” and “R” biotypes. These experiments were performed in a temperature-controlled growth chamber using seeds that had undergone dry storage for between 2.2 and 3 years. At 5°C, seeds from neither *S. arvensis* “R” and “S” biotypes germinated (data not shown). However, at both 10°C and 15°C the maximum cumulative germination was clearly advantageous in favor of “R” compared to “S” biotypes, being, respectively, 48.2–63.8% (10°C) and 50.0–53.2% (15°C) in “R” biotypes vs. 15.9–17.5% (10°C) and 29.1–30.7% (15°C) in “S” biotypes (Figures 5A,B; Supplemental Figure 1).

### Differential seed germination of “S” and “R” *S. arvensis* biotypes is not a question of seed viability or weight difference

**Figure 6:**
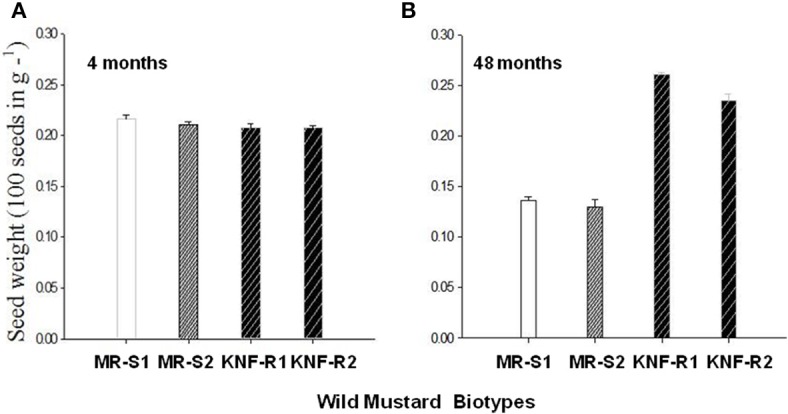
**Mean seed mass of *S. arvensis* susceptible (MR-S1 and MR-S2) and resistant (KNF-R1 and KNF-R2) biotypes after 4 months (A) and 4 years (48 months) (B) of dry storage**. Vertical bars represent standard error (*n* = 3; 100 seeds in 3 batches from each of the four biotypes were weighed).

Seed weight of *S. arvensis* “S” and “R” biotypes measured after dry storage (after-ripened) of 4 months and 4 years are given in Figures 6A,B, respectively. The 4-months dry stored seeds from both lines had similar weight. However, seeds of “S” biotype dry stored for 4 years weighed lesser than the 4-month stored seeds (compare Figure 6B with A, MR-S1 and MR-S2) whereas during the same duration in dry storage, the “R” biotypes weighed more, being significantly heavier than their “S” counterparts (Figures 6A,B).

**Figure 7:**
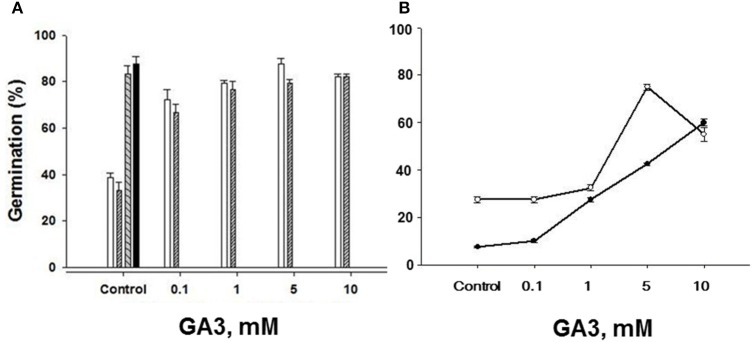
**Gibberellic acid (GA_3_) application restores seed germination potential in chlorsulfuron-susceptible *S. arvensis* biotypes**. **(A)** MR-S1 (open bars) and MR-S2 (serrated bars). Germination (%) of chlorsulfuron-resistant seeds (KNF-R1 and KNF-R2) is shown in “Control” next to the “S” biotypes. Seeds used in **(A)** had been dry stored for 3 years. In **(B)** the response curves to increasing concentrations of GA_3_ are shown with seeds of MR-S2 (■) and KNF-R2 (□) *S. arvensis* biotypes dry stored for 8 years. Vertical bars represent standard error (*n* = 3).

To further ensure that the seeds of both “S” and “R” biotypes were viable, we tested the effects of a range, low to high concentrations, of the plant hormone gibberellic acid (GA_3_) on germination of seeds stored for 3 and 8 years. GA_3_ application to “S” biotype seeds dry stored for 3 years effectively increased their ability to germinate, raising it from 38.8% without GA_3_ to ~80% and higher at 1–5 mM GA_3_ reaching % germination value close to the untreated “R” biotype seeds (Figure 7A). Since we had the “S” and “R” biotypes in dry storage for 8 years, we also compared the effects of GA_3_ on them as well, especially because we had noted a decreasing trend in the germination of even the “R” biotype seeds dry stored for 4 years (see Figure 3C). By 8 years of dry storage, the germination rate of untreated seeds of MR-S2 biotype and KNF-R2 biotype was ~8 and ~26%, respectively. Both these biotypes responded to GA_3_ treatment by vigorous germination that increased with each higher dose of the GA_3_ (Figure 7B). The concentration of GA_3_ required to generate maximal effect on germination potential in these biotypes was found to vary, 5 mM was saturating for “R” biotype while 10 mM was optimal for the “S” biotype—the curve for “S” biotype, however, had not reached saturation (Figure 7B).

## Discussion

Wild mustard (*S. arvensis* L.) is a prolific weed in wheat fields, causing substantial yield loss to the crop. Field management for *S. arvensis* has involved the use of chlorsulfuron herbicides that inhibit acetolactate synthase (ALS) and impair the synthesis of three prominent branched-chain amino acids—valine, leucine, and isoleucine (as stated in the Introduction). However, resistance to this herbicide among weeds has been observed worldwide and generally attributed to amino acid substitutions in the ALS gene (Guttieri et al., 1992; Boutsalis et al., 1999; Gressel, 2002). Weed populations exposed to single-mechanism based herbicides that have longer residual life in the soil develop resistance and, in turn, can incur a fitness cost (Coustau et al., 2000; Tranel and Wright, 2002). The chlorsulfuron-resistant biotypes studied here originated from areas in Turkey, where wheat is mainly grown as a monoculture or in rotation with sunflower. These fields had a history of several years' consecutive use of the chlorsulfuron herbicide. We show and confirm that resistance to chlorsulfuron [ALS (AHAS)-inhibiting herbicides] in Turkish biotypes is associated with altered target site mutations in the ALS gene, corresponding to amino acid substitutions, Pro197 (CCT) to Ser (TCT), or Thr (ACT), as is known for many other species. These findings are consistent with our *in vitro* experiments showing that ALS enzyme activity in *S. arvensis* KNF-R1 and KNFR-2 biotypes, and not the susceptible biotypes, is insensitive to chlorsulfuron. The Pro to Ser (ALS from KNF-R1 biotype) or Thr (ALS from KNF-R2 biotype) substitutions are consistent with previously reported Pro substitution to Ser in Corn poppy (*Papaver rhoeas* L.) (Prado et al., 2004) and wild mustard (*S. arvensis* L.) (Warwick et al., 2005), Ala, Arg, Gln, Leu, Lys, Met, Ser, Thr, or Trp in Kochia (*K. scoparia* L.) (Guttieri et al., 1995; Warwick et al., 2008), or Ala/Thr in wild radish (*R. raphanistrum* L.) (Tan and Medd, 2002).

The newly evolved chlorsulfuron-resistant *S. arvensis* biotypes with mutated ALS gene became a resource to delineate effects on seed germination and weight in comparison to the susceptible biotypes. Interestingly, the time in dry storage (after-ripening) had a major and differential effect on the germinability of seeds from susceptible vs. resistant *S. arvensis* biotypes. The maximum cumulative germination was found to be different between “R” and “S” seeds after dry storage periods from 4 months to 4 years. Seeds from both biotypes held for 4 months in dry storage showed the “S” biotype seeds to record a higher germination than the seed from “R” biotype. However, as the dry storage period was prolonged the germinability of S biotype seeds sharply decreased with longer storage time. Surprisingly, the seeds from “R” biotypes recorded highest germination potential at 24 months dry storage, which slightly dropped at 4 years of dry storage and yet was significantly higher than the “S” biotype seeds. These data on variable germinability of *S. arvensis* being linked to the different biotypes may explain the diverse data on seed germination of this species in the literature. For example, a study on *S. arvensis* seeds immediately after harvest found quite low (7.2%) germinability compared to 28% in those stored for a year (Donald and Hoerauf, 1985), while in another study (Andersson and Milberg, 1998) *S. arvensis* seed germination varied as much as 20–90%. Although it is known that seed dormancy determines germination timing, little is known about how this process is regulated (Xiang et al., 2014).

Differential seed germinability between “S” and “R” biotypes was not due to differential viability, temperature regimen, or non-responsiveness to pro-germination hormone GA_3_, and likely associated with the “R” biotype phenotype due to single amino acid substitution, Pro197 (CCT) to Ser (TCT) or Thr (ACT), in the ALS gene. Thus, while the *S. arvensis* seeds dry stored for 4 months weighed the same irrespective of being sensitive (“S”) or resistant (“R”) to chlorsulfuron, however, after dry storage for 4 years the “R” seeds were significantly heavier than the “S” ones. Interestingly, seed weight and seed germination potential of “S” biotypes apparently correlated—as the seed weight was lost, the germination capacity also decreased. The “R” biotype seeds showed higher germination capacity at 10 and 15°C than the seeds of “S” biotype. This observation bodes well for the fitness of “R” biotype seeds since wild mustard germinates in two flushes, one in spring and the other in autumn both periods of cooler temperatures (Edwards, 1980).

Final evidence for the viability of “S” biotype seeds came from the response pattern of their germination upon application of GA_3_. GA_3_ treatment of “S” biotype seeds dry stored for 36 months resulted in high germinability, reaching a capacity near to that seen with untreated “R” biotype seeds. A comparison of germinability of “S” and “R” biotype seeds dry stored for 8 years demonstrated similar response to exogenous GA_3_, the “R” biotype seed germination saturating at 5 mM GA_3_ while the “S” biotype seeds showed a linear response which appeared to lead them to match the potential of the “R” seeds. Application of the GA_3_ antagonist in seed germination, abscisic acid (ABA), to “R” biotype seeds inhibited their germination (data not shown), suggesting that the germination differences between the two types of seeds may be due to differences in the endogenous ratio of ABA/GA_3_. In preliminary studies, ABA concentrations in the seeds of MR-S1 and MR-S2 *S. arvensis* biotypes were found to be 143–237% higher than the corresponding “R” biotype seeds (unpublished data). However, this remains to be further established. Thus, the ABA/GA hormone postulate (Karssen and Laçka, 1986) might apply to the differential seed germination between “S” and “R” biotypes of *S. arvensis*. In this regard, it is known that GA application is necessary to break dormancy in wild mustard germination (Karssen and Laçka, 1986; Duran and Retamal, 1989). These results are consistent with the findings on chlorsulfuron-resistant lettuce (*Lactuca sativa* L.), where the “R” near-isogenic line showed faster germination than the “S” line (Mallory–Smith et al., 1992), and on *Bromus tectorum* “R” biotypes, which germinated earlier than the “S” biotypes at low temperature (5°C); however, no differences in germination occurred at higher temperatures (Park et al., 2004).

The observation that the *S. arvensis* “R” biotype seeds were significantly heavier than those from the “S” biotypes upon dry storage for 4 years might seem intriguing. However, in this regard it is interesting to note that recent molecular studies with “dry” quiescent seeds showed the latter to be metabolically active as revealed by transcriptomics and protein dynamics (Leubner-Metzger, 2005). Surprisingly, dynamic processes within “dry” seed seem to characterize germination potential, with after-ripening being associated with specific gene networks (Carrera et al., 2008). Further, there is sparse information on post-transcriptional regulation and post-translational modification of proteins in dry seeds, which need to be addressed to throw light on the (molecular) mechanisms that regulate after-ripening (Holdsworth et al., 2007).

Studies on the environmental factors on seed germination/emergence are important for predicting when weeds in the growing season might emerge in order to put an effective weed management in place. To improve their competitive ability in agricultural farms, it is thought that annual weeds adjust the timing and rate of seed germination (Martinez-Ghersa et al., 2000). The degree of competition between the weed and the crop is dependent upon the relative time of their emergence, whichever germinates or emerges first is expected to have the upper hand by employing more time to establish and gainfully compete for space, light, and nutrients over the other (Neve et al., 2003).

The Turkish chlorsulfuron-resistant *S. arvensis* biotypes studied here have the ability to germinate faster in high percentages at cold temperature regimes, and sustain their germinability over a long dry storage time compared to their susceptible counterparts. It would imply that these resistant weeds would be more competitive in the field conditions compared to “S” biotypes. Rapid germination at low temperatures may be a characteristic trait associated with ALS target site resistance (Pro-197/-Ser/-Arg/-Thr), which was postulated to turn into either a fitness advantage or disadvantage depending upon the prevailing agroecological conditions (Park et al., 2004). Moreover, the rapid and high rates of seed germination of chlorsulfuron (ALS inhibitor) resistant *S. arvensis* “R” biotypes could also be “successfully” exploited in weed management strategies to safeguard the crop by draining the “R” weed seed bank. Relative fitness studies on other weeds have shown that the ALS “R” biotypes are not associated with growth penalty, rather under some conditions they may have fitness advantages compared to “S” biotypes (Gressel and Segel, 1990; Maxwell et al., 1990; Thomson et al., 1994; Christoffoleti et al., 1997). Data presented here on comparative seed germination between “R” and “S” biotypes of *S. arvensis* suggest that the altered target site trait conferring resistance to ALS inhibitor, chlorsulfuron in particular, in “R” biotypes does not result in a growth penalty.

### Conflict of interest statement

The authors declare that the research was conducted in the absence of any commercial or financial relationships that could be construed as a potential conflict of interest.
