# Supplementary material for: Seed dormancy is modulated in recently evolved chlorsulfuron-resistant Turkish biotypes of wild mustard (Sinapis arvensis)
Source: Front Chem. 2015 Jul 24;3:46. doi: 10.3389/fchem.2015.00046 (PMC4513236; doi:10.3389/fchem.2015.00046)

Supplemental Figure 1

Cumulative seed germination (%)of chlorsulfuron-resistant ‘R’, KNF-R1 and KNF-R2, and susceptible ‘S’, MR-S1 and MR-S2 *S. arvensis* biotypes sowed in a growth chamber held constant at 10 ºC (A) and 15 ºC (B). Figure shows maximal seed germination on 21 day at the indicated temperature. Vertical bars represent standard error (n=6). See also Figure 5

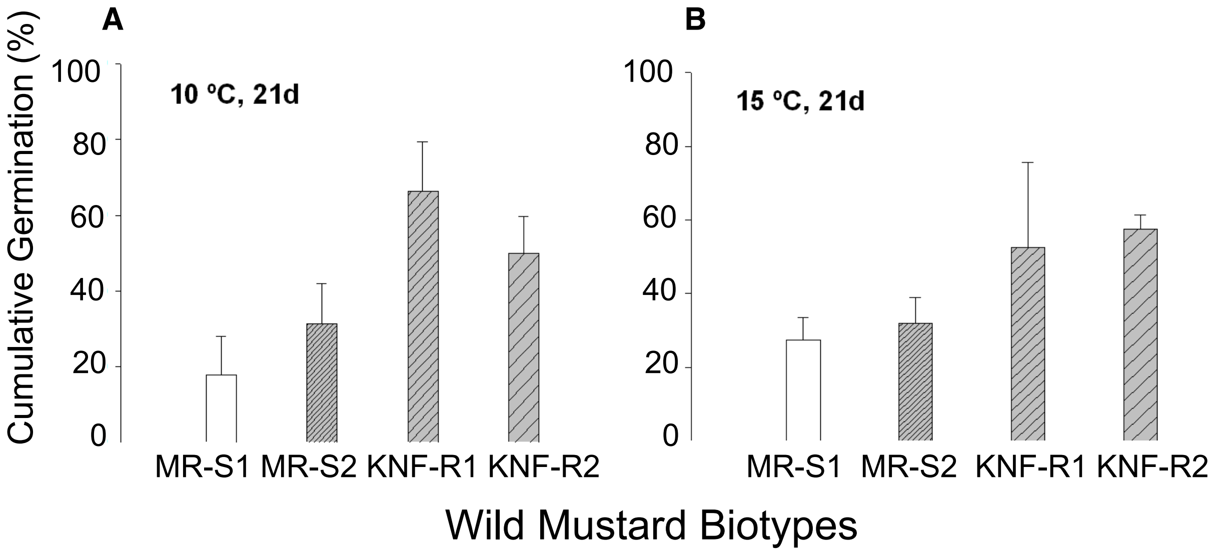

Supplement: Supplementary file 1 [file Presentation1.PDF]
